# An Innovative Method of Obtaining Herbal Propolis Extracts and Application of its Spray-Dried Form as Natural Food Preservatives

**DOI:** 10.1155/ijfo/5546802

**Published:** 2025-06-28

**Authors:** Michał Miłek, Ewelina Sidor, Radosław Bonikowski, Dorota Grabek-Lejko, Gabriela Kowalska, Justyna Rosicka-Kaczmarek, Ewa Ciszkowicz, Małgorzata Dżugan

**Affiliations:** ^1^Department of Chemistry and Food Toxicology, University of Rzeszów, Rzeszów, Poland; ^2^Institute of Natural Products and Cosmetics, Łódź University of Technology, Łódź, Poland; ^3^Department of Bioenergetics, Food Analysis and Microbiology, University of Rzeszów, Rzeszów, Poland; ^4^Institute of Food Technology and Analysis, Łódź University of Technology, Łódź, Poland; ^5^Department of Biotechnology and Bioinformatics, Rzeszów University of Technology, Rzeszów, Poland

**Keywords:** extraction, food preservative, Herbes de Provance, polyphenols, propolis, volatilome

## Abstract

An innovative method of production of the herbal propolis extract (PE) with a masked scent of propolis suitable for food applications was proposed. The obtained extracts based on propolis and selected dried herbs were compared in terms of antioxidant activity, the polyphenolic profile (HPLC), and the volatile fraction (GC × GC-MS, olfactometry), as well as antibacterial activity against *Streptococcus* spp. using serial microdilution method. Based on the highest content of polyphenols and richer volatile fraction, pure PE and Herbes de Provance propolis extract (HPPE) were fractionated by vacuum concentration, obtaining two fractions: hydrophilic and hydrophobic which showed differing antimicrobial effects. Full extracts and fractions were powdered by spray-drying. The positive effect of herbs additive on the powder morphology was confirmed by scanning electron microscopy (SEM) analysis. Good retention of phenolic and volatile components in the obtained powders was demonstrated; however, the composition of powdered fraction was different. Although the antimicrobial activity of powders was not observed in vitro, the first effective attempts were made to use full extracts both in liquid and powdered forms to extend the shelf life of chicken breasts and apple juice. HPPE compared to PE extract turned to be more effective as a preservative, so the proposed method can increase the use of propolis in food preservation.

## 1. Introduction

Trends in healthy eating and clean labeling determine the search for new methods of food preservation that eliminate chemical preservation. Various natural products are being tested, especially plant extracts; for example, rosemary extract has already been officially recognized as a food additive that extends the shelf life of products. Among natural products, propolis seems to be particularly promising, as it has extraordinary antimicrobial properties.

Propolis, also known as bee glue, is a wax-like natural resinous material produced by bees by mixing plant resins with bees' saliva, wax, and pollen to produce the substance which is utilized by honeybees as construction and insulating material as well as a protective coating for the inner walls of the beehive to protect the hive from microbial growth [1, 2]. Among bee products, propolis has been extremely intensively applied as an effective antiseptic, anti-inflammatory, antioxidant, antibacterial, antimycotic, antifungal, antiulcer, anticancer, and immunomodulatory agent in both alternative and traditional medicine [3, 4]. Although propolis is generally considered safe, adverse effects such as dermatitis may be observed in some people [5]. It is indicated that humans can consume 1.4 mg/kg body weight/day or approximately 70 mg/day as a safe dose [6].

The composition of propolis varies depending on the plant source, mainly on tree species from which leaf buds were collected [7]. Thus, diverse types of propolis owing to their color and geographical region were distinguished. In Central Europe, including Poland, bees collect secretion mainly from buds of poplar (*Populus* sp.) and alder (*Alnus* sp.) [8]. More than 400 various propolis components were identified, among which phenolic compounds, mostly flavonoids, phenolic acids, and their esters, are dominant [9, 10]. For poplar Polish propolis, the occurrence of cinnamic and benzoic acid derivatives as well as numerous flavonoids (pinocembrin, pinobanksin, chrysin, and galangin) is characteristic [11–13]. These compounds owning polar properties were proven as antimicrobial components of aqueous and ethanolic propolis extracts (PEs) [14].

Due to its antimicrobial and antioxidant activities, propolis as a natural bioproduct was investigated in food preservation [15, 16]. Many pathogenic bacteria found in food, including *Escherichia coli*, *Listeria monocytogenes*, and *Staphylococcus aureus*, are susceptible to PE [8, 17]. On the other hand, its antioxidant properties delayed the oxidation of lipids and protein which limited negative changes of nutritional food value. Moreover, propolis contributes to the physical and chemical properties of food, thereby maintaining the quality of food during storage [14]. Thus, propolis has excellent potential for extending the shelf life and improving the quality of several food products [14, 16, 18].

However, the applications of propolis as food additives are limited by the low oral bioavailability, bitter taste, and intensive flavor [14, 16]. Due to poor solubility in water, raw propolis has to be extracted by solvent which in turn forces concentration of active ingredients in extracts. Because of its low wax content and high content of bioactive compounds, primarily ethanol or water/ethanol for extraction of propolis is used. As raw propolis among bee products is especially susceptible to pollution, the raw material or extract should be controlled in terms of heavy metals and pesticides [14, 16]. Various applications of PE have been tested, including direct addition to food products (milk or juices) or superficial administration through immersing foods in extract or applying it as a component of protective layer formed on food surfaces (vegetables, fruits, eggs, and meat) [18]. Moreover, cellulose-based packaging materials, which contain additional chitosan/propolis complex, have exciting capabilities for the food packaging sector [19].

Due to the specific organoleptic properties of PE adversely affecting the product's sensory qualities, numerous techniques, such as encapsulation, film casting, and composite materials, were tested to enhance the propolis availability for food applications [14, 16, 20–22]. Among them, spray-drying with various carriers was applied to transform PEs suspended in water into powder form. As spray-drying may decrease strong taste and aroma, this technique has a great potential to increase the application of propolis in the food sector [16, 23]. Propolis encapsulation by spray-drying protects its bioactivity and widen the dosage by water-soluble encapsulation matrix. Good results have been obtained with the use of maltodextrin (alone or in combination with other carriers) as a spray-drying agent produces a dense microcapsule skin and protects the core ingredients against oxygen transfer and possible deterioration [24–26]. Transforming extracts into powder form increases their stability and facilitates dosing in the food industry. There are known cases of successful use of spray-dried PEs in the food industry to create new preservatives with antioxidant properties [16].

The aim of this study was to produce herbal PEs with altered flavor and to convert them into a convenient and stable powdered form by spray-drying. The maintenance of antioxidant and volatile components in raw extracts and powders was evaluated, and the potential of prepared biopreservative with masked propolis smell in food storage durability was tested. Designed preparation with the desired sensory properties, through the selection of a proper masking plant component, could be a natural replacement for synthetic preservatives in the food industry.

## 2. Material and Methods

### 2.1. Raw Materials and Chemicals

Propolis was purchased from a local apiary (Sieklówka, Poland), thyme and Provençal herbs were purchased from Kamis (Stefanowo, Poland), and dried nettle leaves from Kawon (Krajewice, Poland). DPPH (2,2-diphenyl-1-picrylhydrazyl), TPTZ (2,4,6-tris(2-pyridyl)-s-triazine), ABTS (2,2′-azino-bis(3-ethylbenzothiazoline-6-sulfonic acid) diammonium salt), N,O-bis(trimethylsilyl)trifluoroacetamide, sodium carbonate, aluminum chloride, Folin–Ciocalteu reagent, and polyphenol standards: caffeic acid, ferulic acid, benzoic acid, *p*-coumaric acid, pinobanksin, sakuranetin, rosmarinic acid, luteolin 7-glucoside, Tryptone Soya Yeast Extract Broth ISO 11290-2:1998, and Tryptone Soya Yeast Extract Agar were purchased from Sigma-Aldrich (Saint Louis, MO, United States). Solvents, ethanol and acetonitrile, were of HPLC grade, obtained from Honeywell Research Chemicals (Charlotte, NC, United States). Plate count agar (PCA), potato dextrose agar (PDA), Mueller–Hinton agar (MHA), and Mueller–Hinton broth (MHB) were purchased from Biomaxima (Lublin, Poland).

### 2.2. Novel Propolis Extraction

Propolis extraction was performed based on an original legally protected method [27]. In detail, extracts from commercially available dried herbs (thyme, nettle, and Herbes de Provance) were prepared as follows: 10 g of ground herbs was poured with 100 mL of 70% ethanol in a flask, and the flask was closed with a stopper and subjected to extraction in an ultrasonic bath (Sonic 10, 40 kHz, 20 min, max. final temperature 42°C). The samples were filtered through a filter paper. The separated solution was used as an extractant for subsequent propolis extraction. Ten grams of crushed propolis was poured with 100 mL of herbal extract and shaken in a laboratory shaker for 1 h (Benchmark, 400 rpm). Then, maceration was carried out for 5 days (120 h) in the absence of light, shaking once a day. After the designated time, the samples were filtered through a filter paper. In parallel, PE was prepared analogously using 70% ethanol.

The extracts were concentrated by evaporating the ethanol using a rotary evaporator (VC 2–18 CDPlus, Martin Christ, Osterode am Harz, Germany). The two fractions obtained in this way were subjected to further analysis.

### 2.3. Spray-Drying Method

A water–ethanol suspensions subjected to spray-drying contained raw PE (or 10-fold diluted both concentrated fractions), water, and low-saccharificated maltodextrin (Novamyl, Poland) as carrier in the ratio of 10 mL:100 mL:30 g. Powder for herbal PE was obtained in an identical manner.

The mixtures were stirred on a magnetic stirrer for 22 h at room temperature (20°C), to cross-link the carrier fraction [28]. Spray-drying was carried out using a Buchi B-290 dryer with the following drying parameters: inlet temperature 110°C, outlet temperature 80°C, and flow velocity 3.5 mL min^−1^.

### 2.4. Microscopic Evaluation of Powders

The analysis of the powder microstructure was performed using the scanning electron microscopy (SEM) technique. The SEM images were recorded using a JEOL JCM-6000 microscope (JEOL, Akishima, Tokyo, Japan). The tested samples were attached to the microscope stage using the bulk method with self-adhesive graphite discs. Then, the samples were covered with a conductive layer, that is, they were sputtered with gold in a vacuum, without the participation of a noble gas. The images were recorded at acceleration potential differences in the range of 5–10 kV.

### 2.5. Polyphenol Content and Antioxidant Properties

In crude herbal and herbal PEs, total phenolic and flavonoid content was determined for 100-fold dilutions. In the case of the concentrated lower fraction, an additional 10-fold dilution with 70% ethanol was used. Powders were analyzed as ethanol (70%) extracts at a concentration of 100 mg mL^−1^ after thorough mixing on vortex and centrifugation (5 min; 10,000 rpm).

Total phenolic content was determined by a modified method of Singleton and Rossi [29], adapted for use in microplates. Briefly, 100 *μ*L of 10-fold diluted Folin–Ciocalteu reagent was added to 20 *μ*L of the sample (in an appropriate dilution) followed by 80 *μ*L of 7.5% Na_2_CO_3_ solution. After an hour of incubation in the dark, absorbance was measured using a microplate reader (Epoch 2, BioTek) at a wavelength of 760 nm. Results were calculated based on a standard curve for gallic acid and expressed as milligram GAEs (gallic acid equivalents) per milliliter of extract.

Total flavonoid content was assessed using a modified method of Biju et al. [30], adapted for use in microplates. Briefly, 100 *μ*L of 1% AlCl_3_ in methanol was added to 100 *μ*L of the sample (at an appropriate dilution). After 10 min incubation in the dark, absorbance was measured using a microplate reader (Epoch 2, BioTek) at 415 nm. Results were calculated based on the standard curve for quercetin and expressed as milligram QEs (quercetin equivalents) per milliliter of extract.

The reducing power of Fe(III) ions was determined by the FRAP method according to Benzie and Strain [31], with adaptation to measurement on microplates. To 20 *μ*L of the appropriately diluted sample, 180 *μ*L of FRAP reagent was added, and after incubation for 10 min at 37°C, absorbance was measured at 593 nm (Epoch 2, BioTek). Results were calculated from the standard curve on Trolox and expressed as micromole TEs (Trolox equivalents) per milliliter of extract.

The DPPH radical scavenging potential was determined according to Blois [32], with adaptation to microplate measurements. To 20 *μ*L of the appropriately diluted sample was added 180 *μ*L of DPPH reagent (0.1 mM in methanol, diluted to absorbance 0.9), and after incubation for 30 min in the dark, the absorbance was measured at 517 nm (Epoch 2, BioTek). Results were calculated from the standard curve on Trolox and expressed as micromole TEs per milliliter of extract.

The scavenging potential of the ABTS cation radical was determined according to Re et al. [33], with adaptation to measurement on microplates. To 20 *μ*L of appropriately diluted sample was added 180 *μ*L of ABTS reagent (0.1 mM in phosphate buffer pH 7.4, diluted to absorbance 0.8), and after incubation for 6 min in the dark, the absorbance was measured at 734 nm (Epoch 2, BioTek). Results were calculated from the standard curve on Trolox and expressed as micromole TEs per milliliter of extract.

### 2.6. GC × GC-MS Volatilome Analysis

Samples of extracts and fractions for chromatographic analysis were filtered through 0.22-*μ*m syringe filters and secured in appropriately labeled glass vials. In the case of powder analysis, the samples were additionally dissolved in 20% NaCl solution.

The headspace volatile compound profile was determined as described in a previous paper by Miłek et al. [34].

Volatile compounds in the extracts were analyzed by direct injection of the extract according to the procedure described above.

Olfactometric analysis was performed using the ODOII olfactometric attachment from SGE Analytical Science integrated with the MEGA gas chromatograph from Carlo Erba. Chromatography condition is as follows: Rtx-1 column (length 30 m, internal diameter 0.25 mm, and stationary phase film thickness 0.25 *μ*m). The column oven temperature program is 60°C (1 min) to 280°C (20 min) with a temperature increase of 6°C/min.

GC × GC-MS chromatographic analysis of extracts after derivatization: To a weighed solid sample of 5 mg, 250 *μ*L of *N*,*O*-bis(trimethylsilyl)trifluoroacetamide was added, and the samples were incubated at 80°C for 1 h. After this time, 500 *μ*L of tert-butyl methyl ether was added, and the samples were analyzed by GC × GC-MS. The analysis conditions, including peak identification, were described in a previous paper by Miłek et al. [34]. The content of volatile compounds is expressed as a percentage of total peak area. The components were identified by comparing their mass spectra (EI+ mode, electron energy 70 eV) with the Wiley Registry/NIST Mass Spectral Library and Food, Flavors, Fragrances, and Related Compounds MS Library.

### 2.7. HPLC Polyphenol Determination

For HPLC analyses, extracts were diluted fivefold (concentrated lower fractions as a 50 mg mL^−1^ solution in ethanol) and filtered through 0.22-*μ*m syringe filters. Analyses were performed using a Gilson chromatographic system (Gilson Inc., Middleton, WI, United States) equipped with a binary gradient pump (Gilson 322), a column thermostat (KNAUER, Berlin, Germany), an autosampler with fraction collector (Liquid Handler GX-271, Gilson Inc., Middleton, WI, United States), and a diode array detector (DAD, Gilson 172, Gilson Inc., Middleton, WI, United States). An analytical column (Poroshell 120, EC-C18, 4.6 × 150 mm, Agilent Technologies Inc., Santa Clara, CA, United States). The column was operated at 40°C. A gradient elution program was used (A: 0.1% HCOOH in water and B: acetonitrile): 10% B (1.5 min), 10%–100% B (1.5–20 min), 100% B (20–25 min), and again 10% B to equilibrate column. Ten microliters of sample were injected. The chromatograms were recorded at 254, 280, 320, and 360 nm. Identification was made based on comparison with standards (caffeic acid, ferulic acid, *p*-coumaric acid, benzoic acid, sakuranetin, pinobanksin, pinocembrin, rosmarinic acid, and luteolin 7-glucoside [Sigma-Aldrich]), and quantification was based on standard curves prepared for these standards (in the range of 25–400 *μ*g mL^−1^). The content of individual compounds was expressed in mg mL^−1^ of extracts.

### 2.8. Antibacterial Effect Assessment

PEs, Herbes de Provance, and Herbes de Provance propolis were evaluated for their activity against *Streptococcus agalactiae* (DSM 2134) and *Streptococcus pyogenes* (DSM 20565) bacteria, originating from the collection of the Department of Biotechnology and Bioinformatics, Faculty of Chemistry, Rzeszów University of Technology. A serial microdilution method was used, and dilutions of the tested extracts were obtained in the range from twofold to 8192-fold. The bacterial culture obtained after 24-h incubation was diluted in Tryptone Soya Yeast Extract Broth to obtain a density of 10^8^ cells mL^−1^ based on optical density (OD600) measurement using a Bio-Rad SmartSpec Plus spectrophotometer. Twofold dilutions of the analyzed extracts and antibiotics were prepared in Tryptone Soya Yeast Extract Broth on sterile 96-well plates, which were inoculated with a culture of certified bacteria at a density of 10^5^ cells mL^−1^. The prepared titration plates were incubated for 24 h under aerobic conditions at 37°C in a MERC laboratory incubator. Then, for each analyzed extract and antibiotic, the minimum inhibitory concentration (MIC) was determined as the lowest concentration inhibiting visible bacterial growth. From the wells for which the MIC was determined and from the wells with two- and fourfold higher concentrations, 20 *μ*L of bacterial suspension was taken and plated under sterile conditions on previously prepared petri dishes with solidified Tryptone Soya Yeast Extract Agar using sterile spreaders. After 24-h incubation, minimal bactericidal concentrations (MBCs) were determined by analyzing bacterial growth in petri dishes.

Powders dissolved in sterile distilled water, DMSO, and 95% ethanol (after the removal of maltodextrin) were analyzed. The study was conducted using *E. coli* ATCC 10536, *Staphylococcus aureus* ATCC 6538, and *S. agalactia*e DSM 20565 strains. The test was performed as described above, and the concentrations obtained were in the range from 0.39 to 50 mg mL^−1^.

### 2.9. Application in Food Preservation–Preliminary Tests

#### 2.9.1. Chicken Breast

The meat was washed, left to drain, and cut into cubes (2 × 2 × 2 cm). Fifty grams of meat was weighed into a beaker, and raw extracts were added (1 mL/50 g): PE (A) and Herbes de Provance propolis extract (B). The meat sample was prepared in the same way by coating the meat pieces with 2.5 g of propolis powder (C) or propolis-herbal powder (D). An untreated meat sample was prepared as a control for A and B, whereas additional control with 2.5 g of maltodextrin for C and D was applied. All samples were mixed thoroughly, sealed with parafilm and stored at 4°C during 5 days. The test was performed in duplicate.

After this time, microbiological tests were performed. For this, 10 g of each sample was collected in sterile homogenization bags, and 90 mL of sterile physiological fluid (0.9% NaCl) was added. Then, the samples were homogenized for 180 s in a homogenizer (BagMixer 400, Interscience, Saint-Nom-la-Bretèche, France). Serial 10-fold dilutions were made for each homogenate and spread onto petri dishes containing PCA for counting mesophilic bacteria and on PDA (for counting yeasts and molds). Plates with PCA medium were incubated for 3 days at 30°C, while plates with PDA medium were incubated at 25°C for 5 days. After incubation, the number of bacterial and fungi colonies grown was calculated. The results are presented as the number of colony-forming units per 1 g of product (CFU g^−1^).

#### 2.9.2. Fresh-Pressed Apple Juice

Three portions (50 mL) of apple juice fresh-pressed in a home juicer were poured into sterile flasks, and 5 g of propolis-herbal powder (A) and 5 g of maltodextrin (B) were added. Juice without additives was left as a control (C). The flasks were left for 72 h at room temperature.

Serial 10-fold dilutions were made from such samples and were surface inoculated onto petri dishes containing PCA for counting mesophilic bacteria and on PDA (for counting yeasts and molds). Plates with PCA medium were incubated for 3 days at 30°C, while plates with PDA medium were incubated at 25°C for 5 days. After incubation, the number of bacterial and fungi colonies grown was calculated. The results are presented as CFU mL^−1^.

### 2.10. Statistical Analysis

All analyses were performed in triplicates, and results are given as mean ± standard deviation. The results were subjected to analysis of variance (ANOVA), and then the significance of differences was assessed using Tukey's test at the significance level of *p* = 0.05. The correlation between the obtained results was assessed using the Pearson test. Statistical processing of results was performed in Statistica 13.3 (StatSoft, Tulsa, OK, United States).

## 3. Results and Discussion

**Figure 1:**
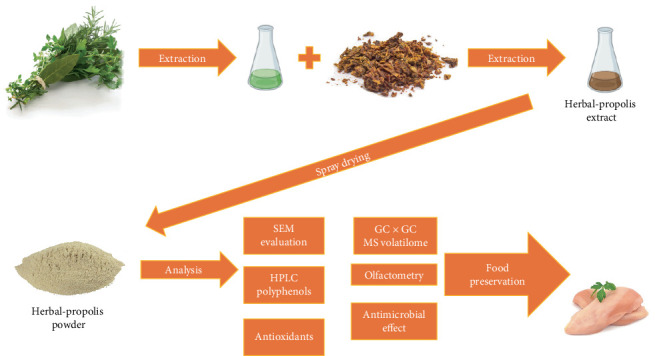
Schematic diagram of the experiment flow.

The conducted research allowed to create an innovative preparation with the smell of propolis masked by herbs for food preservation. The research was carried out in the following stages: (1) production of propolis-herbal extract with the best properties and its vacuum concentration, (2) obtaining a powdered form by spray-drying, and (3) testing the effectiveness of the biopreservative for selected foodstuffs. The process flow is shown in Figure 1.

### 3.1. The Comparison of Prepared Herbal PEs

In the first stage, herbal extracts were prepared and evaluated, which were later used for the subsequent extraction of propolis. Popular herbs were used: thyme and nettle, as well as a popular mixture of herbs, known as Herbes de Provance. This mixture typically contains thyme, sage, peppermint, summer savory, marjoram, and basil [35]. Extracts or essential oils from these herbs are used to preserve meat, also acting as spices [36–38]. Attempts are also being made to extend the shelf life of juices and beverages using herbal extracts and essential oils, including thyme, basil, or peppermint [39–41]. Data on the total content of phenols, flavonoids, and antioxidant capacity determined by the FRAP, DPPH, and ABTS methods were summarized in Table 1.

A strong correlation was observed between antioxidant capacity and the content of polyphenols and flavonoid fractions, regardless of the analytical method used (Pearson *r* coefficients between 0.77 and 0.99). Thyme extract was found to be the richest in phenolic compounds, containing about three times more than Herbes de Provance extract and as much as about 30 times more than nettle extract. Thyme (*Thymus vulgaris*) is one of the most popular spices, known for its high content of phenolic compounds and also a strong antioxidant effect. The values declared by different authors are, however, highly diverse depending on the extraction method of the herbal raw material and the analytical methods used [42]. In the case of Herbes de Provance, as it is a complex herbal mixture, the composition and properties vary depending not only on the extraction method used but also on the composition of the mixture and the quality of the ingredients used.

In the next step, prepared water–ethanol extracts of herbs were used as solvents for subsequent propolis extraction. The extracts obtained were characterized analogously to the initial ones and compared with the pure PE prepared analogously using pure 70% ethanol (Table 1).

Higher content of total polyphenols was noted only in the case of the extract prepared with Herbes de Provance, but the difference was not statistically significant (*p* > 0.05). However, significantly lower flavonoid content was observed in all herbal PEs. This may indicate a certain selectivity of this type of secondary extraction. Using a herbal extract rich in phenolic compounds and other components instead of pure solvent may affect the equilibrium of the extraction process. Another possible explanation for this effect may be the binding of some of the bioactive substances of the herbal extract by the resinous matrix of propolis. Similarly, the lack of a synergistic or even additive effect regarding the content of phenolic compounds was observed in the case of propolis extraction using barberry extract [43]. Simultaneously, this extract exhibited the highest antioxidant activity in all performed tests. It was unexpected, as the initial herbal extract was weaker than the thyme extract. The enhancement of action was not large, reaching approximately 10% in the case of the total phenolic content parameter. The highest increase was observed in the case of the DPPH method, by about 58% compared to the pure PE. A significant decrease in reducing properties was observed only in the case of nettle PE, in the FRAP method. Nettle is known to be rich in iron [44], which may influence ionic interactions in this method of assessing antioxidant properties.

As the odor of obtained propolis-herbal extracts differed in organoleptic analysis, all of them were analyzed for volatile aromatic compounds in comparison to pure PE using the GC × GC-MS method (Table 2).

The profile of volatile compounds of PE was typical for poplar propolis from this region of Europe [13, 45, 46]. It was dominated by isomers of coumaric acid and *p*-vinyl guaiacol. As a result of subsequent propolis extraction with herbal extract, new volatile compounds were introduced, influencing the organoleptic characteristics of the preparation, for example, thymol, carvacrol, and guaiacol. Thymol and carvacrol are substances typical of spice plants such as thyme, oregano, sweet basil, black cumin, and savory. These compounds have a food preservative effect and have a proven positive effect on the human body [47]. Guaiacol has also been shown to have beneficial effects, including antifungal effects [48]. Its presence may also have a positive impact on food preservation applications.

In search of the odor characteristics of the obtained herbal PE, they were subjected to olfactometric analysis in comparison to the initial herbal extracts to determine the aroma profile of the preparations and their suitability for use in food processing.  Table 3 presents the relative intensity of the smell on a scale of 1–10 (1 the least intense to 10 the most intense). As a result of the analysis, it was found that the determinants of the smell of the extracts were: for propolis, vanillin and *p*-vinyl guaiacol; for thyme, thymol, carvacrol, and *p*-vinyl guaiacol; and for herbes de Provance, thymol and carvacrol. In propolis-herbal extracts, the presence of thymol and carvacrol was also found, which gives a herbal, spicy smell. Among the tested, HPPE was characterized by the most complex odor profile. Although the tested fragrance notes were not characterized in the case of nettle extract, its use for propolis extraction made it possible to intensify the existing ones.

As the HPPE extract had the most diverse volatile fraction abundant in active components (thymol, carvacrol, and guaiacol) and at the same time the most favorable antioxidant properties, this PE was selected for further testing.

### 3.2. Separation of HPPE Fractions During Concentration

As a result of ethanol removal from crude extracts during vacuum evaporation, two extract fractions were obtained: the upper one, well miscible with water, and the lower one in the form of a resinous substance, insoluble in water and soluble in ethanol. Both phases were analyzed for polyphenol content and antioxidant activity (Table 4), and selected compounds were identified and quantified using the HPLC-DAD method (Table 5).

It has been observed that in the case of PE, most of the phenolic substances pass to the lower fraction, which is also confirmed by the higher antioxidant capacity. In the case of the combined extract, Herbes de Provance propolis, the relationship is reversed, which results from the different nature of the compounds present in the herbal extract.

Chromatographic analysis showed that in the upper, aqueous phase, only compounds from the phenolic acid group (caffeic, ferulic, *p*-coumaric, and benzoic acids) were present, while flavonoids were concentrated in the lower phase (Table 5). The use of the herbal extract in subsequent propolis extraction resulted in the introduction of additional polyphenols, mainly rosmarinic acid (in high concentration) and some flavonoids, among which luteolin 7-glucoside was identified. Rosmarinic acid is a characteristic secondary metabolite of spice plants, especially those from the Lamiaceae family [49]. This compound possesses strong antimicrobial properties [50, 51].

In order to determine how the composition of the fraction affects the effectiveness of bacteria inhibition, full extracts and isolated fractions were tested against two strains of *Streptococcus* sp. (Table 6).

PE was shown to have a strong effect on both strains, however, twofold higher against *S. agalactiae*. In addition to inhibiting bacterial growth, bactericidal activity was also demonstrated. Interestingly, the propolis-herbal extract inhibited bacterial growth in the same dilutions as the PE alone, but the determined MBC values indicate changes in the mechanism of action. Lower concentrations of both extracts exhibited a lethal effect against *S. pyogenes* compared to *S. agalactiae*. As expected, a stronger antibacterial effect of the lower fraction of the propolis-herbal extract was noted. Hence, it can be concluded that the mechanism of antimicrobial action is mainly determined by flavonoids, which are present in the majority in this fraction.

Propolis has previously been shown to have a strong effect on *Streptococcus* sp., among which the most important pathogenic species are *S. pyogenes* and *S. agalactiae* strains, used in our experiment [52, 53]. *S. pyogenes* is adapted to the human host and cause most often streptococcal pharyngitis, also known as strep throat [54, 55], but also pyoderma, scarlet fever, or invasive diseases, with potential to disrupt both innate and adaptive immune responses to infection. Propolis is reported to interfere with the division of *S. agalactiae*, cytoplasm disruption, and protein synthesis, causing bacterial lysis [56]. Therefore, the higher bactericidal properties of HPPE compared to the PE are a very promising result, indicating potential for developing treatments for infections.

Moreover, *Streptococcus* bacteria are also involved in various food spoilage processes, including undesirable fermentation of dairy products and deterioration of meat products [57, 58]. Other species of the streptococcus genus (e.g., *Streptococcus equi*) may be problematic if contaminated unpasteurized milk or other dairy products are consumed [59]. In the case of meat, this type of bacteria may come from the microflora of the animal's lymph nodes [60]. For this reason, the activity of herbal PEs is also promising for use in food preservation.

### 3.3. HPPE Converting Into Powdered Form

**Figure 2:**
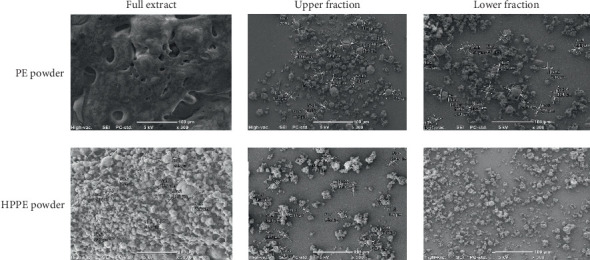
SEM images of the microstructure of the obtained powders (magnification 300×), marker: 100 *μ*m.

Both PE and herbal propolis extract (HPPE) in their full form and as the upper and lower fractions obtained during the concentration of the extracts were subjected to spray-drying. Micrographic analysis of the obtained powders showed their diverse morphological structure, depending on the microcapsule core (Figure 2).

The surface morphology of both the PE and the HPPE powders was significantly different from that of the other preparations. The particles of full extract, especially PE powder, were stuck together and were characterized by liquid bridges, indicating a form of conglomerates; for the other powders, the capsule microstructure was more desirable. All the particles of the obtained powders' upper and lower fractions had a spherical shape, with a heterogeneous surface and comparable sizes. The average particle size of the obtained fixed preparations was 15.3 *μ*m. The amorphous state of the material was confirmed by the smooth, wrinkled capsules with no rough surfaces. The observed surface roughness may be the result of the crystallization of maltodextrin on the capsule surface during spray-drying. Literature data indicate a variable morphology of microcapsules loaded with PEs, depending on the carrier used and drying conditions [25, 61, 62]. However, spray-drying of combined herbal PEs seems to be more beneficial for the morphology of the obtained preparation in this case.

The obtained powders were assessed for their phenolic content and antioxidant activity (FRAP) (Table 7).

The highest polyphenol content and reducing power were found for powders obtained from the upper fractions of the extract, followed by full extracts and the lowest for powders from the lower fractions. The beneficial effect of enriching the extract with Herbes de Provance ingredients was demonstrated for all powders from solid HPPE and its fractions. The obtained values correlate with the data for the initial solutions subjected to spray-drying (data not shown). The result obtained for propolis powder is similar to that reported by Busch et al. [24] for a similar dried preparation using maltodextrin as a carrier (167 mg/100 g). Much higher results, reaching over 20 g GAE/100 g powder, were reported by da Silva et al. [23], but they applied spray-drying without adding a carrier. It has been previously shown that the polyphenol content and antioxidant activity of propolis powders are strongly influenced by process variables such as extract feed rate and air outlet temperature [63]. The selection of the drying medium also influences antioxidant retention [24, 26]. So far, no attempts have been made to dry propolis-herbal extracts, but, for example, honey with the addition of herbal infusions was dried, which significantly increased the antioxidant potential of the obtained preparations [64].

Since spray-drying (using high temperature) may change the profile of volatile fragrance compounds, a comparative analysis of the composition of the volatile fraction of powders was performed. The odor profile of the powders obtained from full extracts (PE and HPPE) was compared by GC × GC-MS in the headspace using the SPME technique (Table 8). In both powders, mainly compounds from the group of methyl benzene derivatives were detected, although the highest percentage was determined for nonanoic acid. In the case of propolis-herbal powder, the richest profile of aroma compounds was found, and additional aroma components were detected, for example, beta-caryophyllene from the group of sesquiterpene hydrocarbons; terpinolene, a component of parsnip oil; gamma-terpinene; *p*-cymene, specific for thyme; prenyl benzoate; carvacrol; and thymol (compounds characteristic of plants from the Lamiaceae family, e.g., thyme and oregano) [47]. It can be concluded that these compounds come from Herbes de Provance, which are a mixture of plants rich in essential oils (rosemary, basil, thyme, sage, peppermint, savory, oregano, and marjoram). Dissolution of the samples revealed a much richer profile of aroma compounds, thanks to the destruction of the polysaccharide matrix of the carrier (maltodextrin). The addition of herbs to microencapsulated preparations results in the appearance of characteristic plant components of essential oils, for example, estragole, bisabolene, terpineol, camphor, and calamine, which was confirmed by the advanced gas chromatography method GC × GC-MS. The richer volatilome of powders than the initial extract may be due to the fact that the extracts were much more diluted and a large proportion of the compounds were at concentrations below the detection threshold. It cannot be ruled out that some of the compounds were formed as a result of exposure to elevated temperature. In the case of spray-drying of solutions containing essential oils, changes in the volatile profile due to the action of temperature are often observed. Less volatile substances (such as carvacrol or caryophyllene) increase their share in the overall profile, while the content of highly volatile compounds such as *γ*-terpinene, *p*-cymene, terpinen-4-ol, and thymol decreases [65–68].

To our knowledge, powders obtained by drying PEs have not been previously evaluated using the GC × GC-MS technique. There is a report on the encapsulation of PE using *β*-cyclodextrin, where the profile of volatile compounds of the extract subjected to encapsulation was assessed, as well as the encapsulation yield of its individual components (expressed as a percentage of the substance in the obtained complex in relation to the initial extract). The highest encapsulation efficiency was noted for phenolic acids (including ferulic, cinnamic, and phloretic acids), significantly lower efficiency was noted for flavonoids [69]. However, volatile compounds were not evaluated.

Spray-drying has been successfully used to obtain dry extracts from many aromatic herbs. Tomazelli Junior et al. [67] evaluated spray-dried aroma extracted from rosemary and found that this preservation technique caused some decreases in the content of volatile compounds, especially those containing hydroxyl groups, while they observed an increase in the content of several substances, including carvacrol, linalool, and borneol [67]. In the case of spray-drying of savory extract, it was observed that a higher proportion of carrier (maltodextrin) causes a decrease in the content of essential oil components in the powder. This is explained by the fact that maltodextrin reduces the diffusion coefficients for volatile organic compounds [70]. Therefore, the salt solution method we used in sample preparation allowed us to reveal the full profile of volatile aroma compounds in the analyzed powders. On the other hand, masking the odor while maintaining the beneficial properties, especially in the case of propolis, may be beneficial for the use of powders thus obtained in the food industry.

To describe the odors of the obtained powders, they were subjected to olfactometric analysis (Table 9). Propolis powders in undissolved form do not show any smell notes that can be detected by this method. Dissolving the powders in 20% NaCl solution reveals smell note characteristic of vanillin present in propolis, and in the case of powders based on propolis-herbal extract, smell note characteristic of Herbes de Provance (thymol and carvacrol). This phenomenon may be due to the matrix–complexation effect. Firstly, the compounds were released from the encapsulated/complexed form, and secondly, there is a salting-out effect, that is, a significant reduction in the affinity of volatiles for water.

As a contribution to the potential use of propolis-herbal powders in food preservation, their effect on selected bacterial strains was analyzed. *E. coli* is known to be found in raw or undercooked meat products [71] and dairy, especially raw milk and cheeses [72]. Meat and meat products, poultry and egg products, and milk and dairy products are implicated in Staphylococcal food-borne disease [73], and *S. agalactiae* is known to be transmitted by raw fish products [74]. Thus, these strains were included in the research; however, the results (Table 10) indicate that the only inhibitory effect was noted against the *S. agalactiae* strain for the powder from the lower propolis fraction and both fractions of the propolis-herbal extract. The solvent used to dissolve the powder had an impact on its performance, water proved to be the best.

### 3.4. Food Preservation Trials

**Figure 3:**
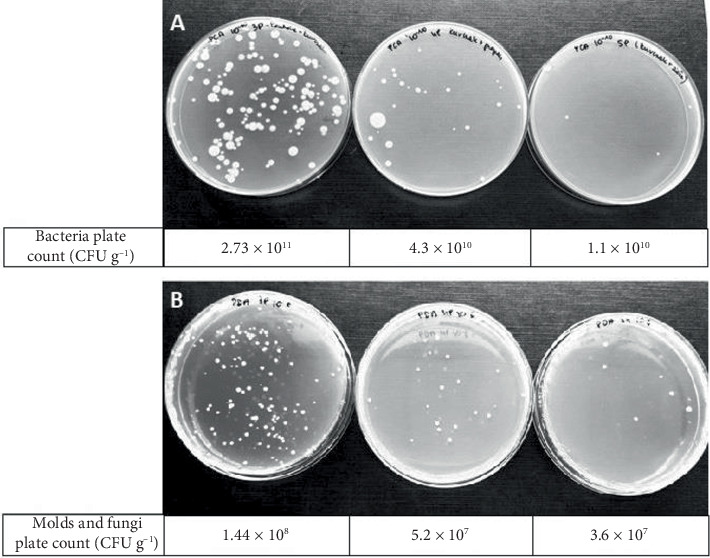
Image of plates after incubation: (A) PCA medium and (B) PDA medium. From left: control sample (without treatment), meat with PE, and meat with HPPE. The values below the plates indicate the number of colonies after inoculation from propolis extract–preserved chicken meat samples in CFU g^−1^.

As good antibacterial activity was confirmed in vitro, liquid Herbes de Provance propolis extract (HPPE) was firstly tried to preserve chicken breast compared to pure PE. The addition of both extracts resulted in a significant reduction in the number of grown bacterial colonies (Figure 3), related to the control sample. For total bacterial counts, a reduction of approximately 1.4 log was observed when HPPE was used, while for yeasts and molds, the reduction was approximately 0.6 log.

**Figure 4:**
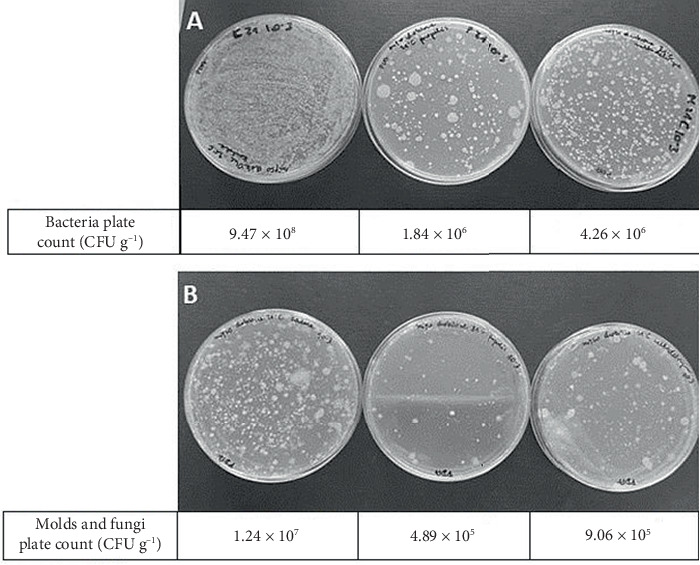
Image of plates after incubation: (A) PCA medium and (B) PDA medium. From left: control sample (without treatment), meat with HPPE powder, and meat with maltodextrin. The values below the plates indicate the number of colonies after inoculation from propolis extract–preserved chicken meat samples in CFU g^−1^.

Although the reduction of antibacterial activity of powder form was observed in vitro, HPPE powder was used to preserve chicken breast (Figure 4). Compared to untreated meat samples, using HPPE powder resulted in significant inhibition of microorganism growth (reduction of 2.71 for bacteria and 1.4 log for yeast and mold, respectively). Maltodextrin itself also showed an inhibitory effect.

The usefulness of using propolis to extend the shelf life of meat products has been previously confirmed by a significant reduction in the number of microorganisms in this type of product. In the case of using ethanolic PE for preserving fermented sausages, it was possible to reduce the number of *Listeria innocua* bacteria by 3 log CFU/g [75]. Also, in the case of sausages, the use of propolis reduced the number of bacteria by about 6 log CFU/g and the number of molds and yeasts by 6.6 log CFU/g [76]. Another example is the extension of the shelf life of beef patties, where a reduction of mesophilic bacteria and psychrophilic bacteria was achieved (by 1 and 3 log, respectively) [77].

#### 3.4.1. Fresh-Pressed Apple Juice

**Figure 5:**
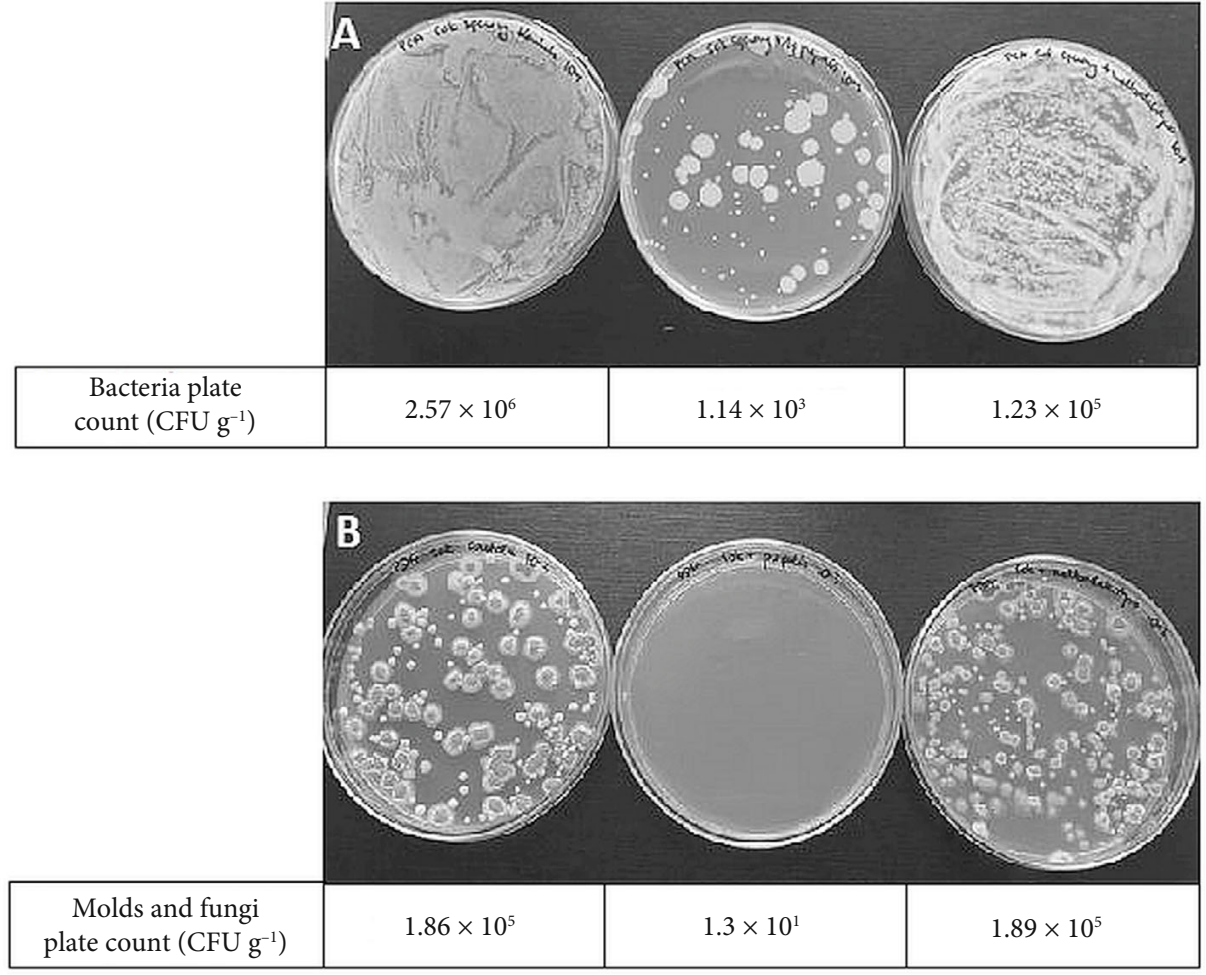
Image of dishes after incubation: (A) PCA medium and (B) PDA medium. From left: control sample (without treatment), juice with HPPE powder, and juice with maltodextrin. The values below the plates indicate the number of colonies after inoculation from propolis extract–preserved fresh apple juice samples in CFU g^−1^.

Similarly, after sowing the samples (1000× diluted) on solid media with PCA, a significant reduction in the number of bacteria was observed in the juice with the addition of HPPE powder (Figure 5a). The addition of maltodextrin itself also inhibits bacterial growth. In the case of the control sample and with maltodextrin, the number of bacterial colonies grown from the dilution shown in the photo is so large that growth in the form of a lawn is observed, while in the juice with the addition of HPPE powder, only single bacterial colonies are visible. On the PDA medium used for the growth of yeast and molds, no growth of yeast colonies is visible in the juice with the addition of HPPE powder (Figure 5b), while in the control sample and the juice with maltodextrin, a large number of fungal colonies are visible. When a lower dilution of the juice was plated (10×), the control plates were completely covered with molds and yeasts, whereas in the case of the juice with HPPE powder, only one colony appeared on the surface of the medium (data not shown).

When PE was added to apple juices, the number of *E. coli* bacteria was reduced by as much as 5 log CFU/mL [78]. In our studies, the reduction was approximately 2.71 log for bacteria and 1.14 log for molds and fungi. It has been repeatedly proven that propolis inhibits the growth of molds and fungi as well as reduces the number of *Bacillus* spores when used to preserve fruit juices [79–81].

The reduction in the number of microorganisms as a result of the preservative action may also be related to the initial microbiological purity of the product. In our experiment for chicken breast, the total bacterial content was below 1 × 10^5^ CFU/g and total molds and yeast was below 1.8 × 10^1^ CFU/g, while for apple juice, the values were below 3 × 10^2^ CFU/g and 4 × 10^2^ CFU/g, respectively.

So far, many attempts have been made to use propolis in preserving various types of food [18]. Propolis-based approaches have also been used to extend the shelf life of fresh juices. Yang et al. [81] found that the addition of propolis emulsion to orange juice inhibited bacterial growth and vitamin C degradation. Luis-Villaroya et al. [82] used PE in combination with mild temperature to preserve apple juice. They obtained inhibition of *L. monocytogenes* and *E. coli* bacterial growth. Meat is also a product that is being tried to be preserved with propolis. Chitosan coatings with 1% or 2% of PE reduced the growth of bacteria from different groups (coliforms, psychrotrophs, lactic acid bacteria, and aerobic mesophilic bacteria) during 12-day storage of chicken breast [83]. Marinating chicken breast with a marinade-containing PE (up to 12% v/w) also reduced the number of microorganisms, including molds and fungi after storage [63]. Attempts are also being made to use propolis to extend the shelf life of beef [84, 85] or fish [15] as well as sausage products [86, 87]. The use of propolis in combination with turmeric reduced the growth of spoilage microorganisms in minced beef [65]. The great potential of propolis for preserving food is well documented, but the authors draw attention to the change in the sensory properties of preserved food [18, 83, 88].

## 4. Conclusion

The use of the addition of herbs' volatile components during the subsequent extraction of propolis allows to mask of typical smell of propolis, which is the main limitation of the use of propolis as a food preservative. Moreover, fractionation of the extract by vacuum concentration allowed to obtain two fractions differing in chemical composition and smell; unfortunately, hydrophilic fractions exhibited low antibacterial activity. When the propolis-herbal extract was transformed into powder by spray-drying, the antioxidant and aromatic properties were preserved, but the antibacterial activity in vitro was completely reduced. However, the first tests confirm that both the full liquid extract and its powdered form can be used to preserve chicken breast and fresh apple juice. This can be explained that if the microcapsule is placed in the nutritional matrix, the bioactive core, which has antimicrobial properties, is slowly released. The use of propolis and herbs synergism made it possible to obtain a natural preservative preparation (in liquid or powder form) with improved organoleptic properties, enhanced antioxidant properties, and good antibacterial activity. Moreover, the preparation can be designed for a specific food product, for example, propolis-thyme extract for meat preservation, propolis-chokeberry extract for fruit juices, or propolis-garlic extract for vegetable juices. The new method was used for the first time for the preparation of herbal PE, and the obtained promising results show a new solution that may increase the use of propolis in the food industry. Admittedly, preliminary laboratory studies have confirmed the effectiveness of propolis-herbal preparations in inhibiting the development of microflora in raw chicken meat and apple juice; however, their use for food preservation in industrial conditions requires further research.

## Figures and Tables

**Table 1:** The content of total phenols and flavonoids and the antioxidant activity of herbal extracts used to subsequent propolis extraction.

	**TPC (mg GAE mL** ^**−1**^)	**TFC (mg QE mL** ^**−1**^)	**FRAP (*μ*mol TE mL** ^**−1**^)	**DPPH (*μ*mol TE mL** ^**−1**^)	**ABTS (*μ*mol TE mL** ^**−1**^)
Herbal extracts					
Thyme extract (TE)	10.02 ± 0.03^c^	2.94 ± 0.03^c^	51.07 ± 4.12^b^	16.42 ± 0.41^c^	71.53 ± 0.00^c^
Herbes de Provance extract (HPE)	3.49 ± 0.01^b^	1.68 ± 0.11^b^	49.18 ± 2.65^b^	6.23 ± 0.18^b^	65.98 ± 1.57^b^
Nettle extract (NE)	0.31 ± 0.01^a^	0.29 ± 0.04^a^	1.43 ± 0.16^a^	0.29 ± 0.04^a^	21.51 ± 0.27^a^
Herbal propolis extracts					
Control propolis extract (PE)	13.34 ± 0.09^AB^	9.98 ± 0.49^C^	52.32 ± 0.63^B^	17.73 ± 0.08^A^	386.47 ± 26.66^AB^
Thyme propolis extract (TPE)	13.24 ± 1.27^A^	5.32 ± 0.29^A^	58.17 ± 2.67^C^	21.23 ± 0.31^C^	406.98 ± 7.06^AB^
Herbes de Provance propolis extract (HPPE)	14.57 ± 0.42^B^	6.21 ± 0.23^B^	57.76 ± 1.95^C^	28.09 ± 0.50^D^	409.20 ± 36.85^B^
Nettle propolis extract (NPE)	12.45 ± 0.35^A^	5.50 ± 0.19^A^	47.34 ± 2.61^A^	19.35 ± 0.08^B^	364.84 ± 2.35^A^

*Note:* Means for herbal extracts marked with different lowercase superscript letters (a, b, and c) in a column are significantly different (*p* < 0.05). Means for herbal propolis extracts marked with different uppercase superscript letters (A, B, C, and D) in a column are significantly different (*p* < 0.05).

**Table 2:** GC × GC-MS profiles of propolis and herbal propolis extracts.

**Compound**	**RI**_**exp.**_	**RI**_**lit.**_	**Extract**			
			**% of total peak area**			
			**PE**	**TPE**	**HPPE**	**NPE**
Phenol	978	980	ND	1.29	ND	1.23
Benzyl alcohol	1039	1043	ND	1.38	0.96	1.18
Guaiacol	1091	1088	ND	3.29	1.71	3.3
Benzoic acid	1165	1159	5.04	4.49	6.49	6.13
1,2,3-Propanetriol, diacetate	1232	1230	ND	3	2.79	ND
Thymol	1294	1290	ND	2.01	0.86	ND
Carvacrol	1302	1300	ND	ND	1.76	ND
*p*-Vinyl guaiacol	1317	1314	11.48	24.14	26.34	16.9
Dihydroeugenol	1357	1352	1.49	0.64	ND	ND
Vanillin	1400	1398	3.61	3.47	3.65	4.78
Guaiacylacetone	1535	1536	ND	1.53	ND	ND
o-Coumaric acid	1552	No data	20.8	32.83	35.02	33.07
Homovanilic acid methyl ester	1717	1714	4.46	2.17	0.55	6.01
Benzyl benzoate	1768	1769	4.12	3.59	5.26	4.71
*p*-Coumaric acid, (trans)	1787	No data	18.59	5.11	2.81	7.52
Ethyl myristate	1798	1795	ND	ND	0.17	ND
2-Phenylethyl benzoate	1858	1859	3.07	0.77	0.73	0.73
Phenylmethyl salicylate	1894	1898	1.47	0.94	1.02	1.66
Benzyl cinnamate	2137	2135	9.15	5.62	4.89	7.54
Ethyl *p*-hydroxycinnamate	2395	No data	5.35	1.97	2.71	2.4
Pinostrobin chalcone	2404	2402	9.67	1.76	2.28	2.84
5-Hydroxy-4′,7-dimethoxyflavanone	2962	2964	1.7	ND	ND	ND

Abbreviations: ND, not detected; RI_exp._, calculated retention index; RI_lit._, literature retention index.

**Table 3:** Results of olfactometric analysis of tested extracts.

	**Smell descriptors**			
	**Vanillin (sweet vanilla, creamy, chocolate)**	***p*-Vinyl guaiacol (clove, phenolic, peppery, smoky, woody, powdery)**	**Thymol (herbal, thyme, camphor phenolic)**	**Carvacrol (spicy, woody, camphor thymol)**
PE	1	2	—	—
TE	—	2	10	6
HPE	—	—	1	4
NE	—	—	—	—
TPE	2	9	6	—
HPPE	3	10	2	2
NPE	3	5	—	—

**Table 4:** The content of total phenols and flavonoids and the antioxidant activity of fractions obtained after concentration of extracts.

	**Full PE**	**PE upper fraction**	**PE lower fraction**	**Full HPPE**	**HPPE upper fraction**	**HPPE lower fraction**
TPC (mg GAE mL^−1^)	13.34 ± 0.09^c^	7.55 ± 0.41^a^	11.3 ± 0.14^b^	14.57 ± 0.42^d^	15.96 ± 0.22^e^	8.33 ± 0.19^a^
TFC (mg QE mL^−1^)	9.98 ± 0.49^d^	0.16 ± 0.00^a^	10.51 ± 0.68^d^	6.21 ± 0.23^c^	3.81 ± 0.16^b^	3.32 ± 0.16^b^
FRAP (*μ*mol TE mL^−1^)	52.32 ± 0.63^b^	28.22 ± 1.77^a^	34.64 ± 3.68^a^	57.76 ± 1.95^b^	73.42 ± 5.49^c^	32.75 ± 1.7^a^
DPPH (*μ*mol TE mL^−1^)	17.73 ± 0.08^b^	10.82 ± 0.3^a^	23.42 ± 0.23^c^	28.09 ± 0.5^d^	32.36 ± 0.57^e^	18.11 ± 0.15^b^
ABTS (*μ*mol TE mL^−1^)	386.47 ± 26.66^c^	513.44 ± 7.06^d^	321.04 ± 20.39^b^	409.2 ± 36.85^c^	370.39 ± 18.04^bc^	233.99 ± 19.6^a^

*Note:* Means marked with different superscript letters (a, b, c, d, and e) in a row are significantly different (*p* < 0.05).

**Table 5:** HPLC quantitative determination of selected polyphenols.

**Compound**	**Full PE**	**PE upper fraction**	**PE lower fraction**	**Full HPPE**	**HPPE upper fraction**	**HPPE lower fraction**
Caffeic acid (mg mL^−1^)	0.085 ± 0.006^a^	0.381 ± 0.026^c^	0.063 ± 0.006^a^	0.083 ± 0.010^a^	0.249 ± 0.013^b^	< LOQ
*p*-Coumaric acid (mg mL^−1^)	2.332 ± 0.020^a^	2.901 ± 0.164^ab^	3.148 ± 0.250^b^	2.786 ± 0.166^a^	4.714 ± 0.226^c^	11.777 ± 0.620^d^
Ferulic acid (mg mL^−1^)	0.759 ± 0.041^a^	0.810 ± 0.062^ab^	1.036 ± 0.107^b^	0.758 ± 0.102^a^	1.284 ± 0.204^b^	2.664 ± 0.225^c^
Benzoic acid (mg mL^−1^)	1.224 ± 0.103^b^	1.177 ± 0.162^b^	0.204 ± 0.034^a^	1.299 ± 0.119^b^	2.075 ± 0.189^c^	5.824 ± 0.351^d^
Sakuranetin (mg mL^−1^)	1.299 ± 0.056^b^	< LOQ	1.462 ± 0.112^b^	1.123 ± 0.085^b^	0.540 ± 0.062^a^	14.134 ± 0.954^c^
Pinobanksin (mg mL^−1^)	0.322 ± 0.030^a^	< LOQ	0.413 ± 0.033^b^	0.307 ± 0.022^ab^	0.208 ± 0.015^a^	2.465 ± 0.099^c^
Pinocembrin (mg mL^−1^)	0.426 ± 0.016^c^	< LOQ	0.463 ± 0.029^c^	0.326 ± 0.016^b^	0.145 ± 0.020^a^	4.654 ± 0.204^d^
Rosmarinic acid (mg mL^−1^)	ND	ND	ND	22.518 ± 0.942^b^	1.838 ± 0.106^a^	51.028 ± 1.564^c^
Luteolin 7-glucoside (mg mL^−1^)	ND	ND	ND	0.032 ± 0.003^a^	ND	0.038 ± 0.006^a^

*Note:* Means marked with different superscript letters (a, b, c, and d) in a row are significantly different (*p* < 0.05).

Abbreviations: LOQ, below the limit of quantitation; ND, not detected.

**Table 6:** Antibacterial effect of propolis and Herbes de Provance propolis extract.

	***Streptococcus agalactiae***		***Streptococcus pyogenes***	
	**MIC (sample dilution)**	**MBC (sample dilution)**	**MIC (sample dilution)**	**MBC (sample dilution)**
PE	4096	1024	2048	512
HPE	8	2	8	> 2^a^
Full HPPE	4096	> 2^a^	2048	1024
HPPE upper fraction	256	> 2^a^	256	> 2^a^
HPPE lower fraction	4096	> 2^a^	1024	> 2^a^

^a^MBC not designated, no bactericidal mechanism of action of extracts in the range of dilutions from 2 to 8192-fold.

**Table 7:** Total phenolic content and reducing power of propolis and Herbes de Provance propolis powders.

**Sample**	**TPC (mg GAE/100 g)**	**FRAP (*μ*mol TE/100 g)**
PE powder	171.13 ± 11.22^b^	582.24 ± 12.41^c^
PE upper fraction powder	237.10 ± 6.31^c^	773.03 ± 12.41^d^
PE lower fraction powder	133.18 ± 3.16^ab^	405.15 ± 5.43^b^
HPPE powder	210.32 ± 1.40^c^	817.98 ± 20.16^d^
HPPE upper fraction powder	348.46 ± 16.13^d^	1442.98 ± 54.27^e^
HPPE lower fraction powder	110.62 ± 2.10^a^	329.95 ± 15.51^a^

*Note:* Means sharing the same superscript letters (a, b, c, d, and e) are not significantly different (*p* > 0.05).

**Table 8:** GC × GC-MS profiles of spray-dried powders.

**Compound**	**RI**_**exp.**_	**RI**_**lit.**_	**% of total peak area**			
			**PE powder**		**HPPE powder**	
			**Crude powder**	**Powder dissolved in 20% NaCl**	**Crude powder**	**Powder dissolved in 20% NaCl**
Ethanol	464	459	43.95	1.25	2.33	0.03
Beta-Myrcene	992	988			0.39	0.06
*p*-Cymene	1016	1020			15.16	1.72
*o*-Cymene	1030	1022	3.50		4.43	0.76
Eucalyptol	1026	1032			6.06	1.90
Gamma-Terpinene	1057	1054			7.69	0.89
1-Ethyl-2,4-dimethylbenzene	1079	1078	1.45			
2,4-Diethyl-1-methylbenzene	1072	1078	3.79			
Isoterpinene	1080	1088			1.09	0.05
Linalool	1098	1095				1.69
Nonanal	1101	1100				0.06
1,3,8-*p*-Menthatriene	1116	1108				0.10
1,2,4,5-Tetramethylbenzene	1114	1118	2.59			
Allocimene	1131	1127				0.46
Isopropyl myristate	1135	1128		0.85		0.12
Camphor	1133	1141				0.37
Benzoic acid	1158	1159				1.19
1,4-Diethyl-2-methylbenzene	1166	1144^a^			1.39	
Borneol	1167	1165				0.02
Ethyl benzoate	1168	1169				0.11
4-Carvomenthenol	1181	1177			1.23	1.04
Alpha-Terpineol	1190	1195				2.18
Estragole	1204	1198			2.78	2.72
Homomyrtenol	1214	1212				3.35
Thymol methyl ether	1230	1232			4.94	0.11
Ascaridole	1242	1234				0.15
Carvacrol methyl ether	1242	1241				0.33
Thymoquinone	1241	1248				1.20
Bornyl acetate	1250	1254				0.10
Nonanoic acid	1269	1267	7.75		2.20	
1,3-Bis(1,1-dimethylethyl)benzene	1273	1249^a^		0.43	1.80	
Thymol	1284	1290			3.12	5.00
Nonanoic acid ethyl ester	1300	1296				0.10
Methyl cinnamate	1303	1299				1.02
Carvacrol	1298	1300			6.86	3.64
2-Methylpropyl benzoate	1317	1311		0.25		0.21
*p*-Vinyl guaiacol	1319	1314				0.16
Terpinyl acetate	1339	1346				1.33
Ethyl 3-phenylpropionate	1357	1356		1.16		1.40
Eugenol	1354	1356				0.56
Alpha-Ylangene	1369	1373		0.49		0.22
Alpha-Copaene	1367	1374		0.53		
(Z)-Ethyl cinnamate	1379	1376				0.19
Ethyl decanoate	1392	1395		0.76		0.16
Vanillin	1393	1398		0.99		0.14
Methyl eugenol	1402	1403				0.31
Beta-Caryophyllene	1411	1417		2.36	1.88	5.14
Trans-alpha-Bergamotene	1439	1432				0.81
Aromadendrene	1444	1439		3.80		3.23
Alpha-Humulene	1448	1452				3.92
Pentyl benzoate	1469	1476		3.45		0.83
Gamma-Muurolene	1483	1478		0.74		1.64
Prenyl benzoate	1484	1485		8.01	1.44	8.27
Alpha-Selinene	1492	1498		1.75		
Alpha-Bisabolene	1501	1506				1.50
Trans-Calamenene	1523	1521				0.10
Delta-Cadinene	1525	1522				0.22
Cis-Calamenene	1532	1528		1.13		0.55
Prenyl salicylate	1556	No data		0.64		0.28
Spathulenol	1580	1577				0.34
Trans-Sesquisabinene hydrate	1569	1577		1.30		
Caryophyllene oxide	1574	1582		3.96		2.59
Ethyl dodecanoate	1602	1594		4.46		3.83
Alpha-Muurolol	1650	1644		2.29		
Tau-Cadinol	1657	1650				0.54
Delta-Cadinol	1645	1651				0.88
6-Deisopropylatrazine	1649	1654				0.08
Cadalene	1670	1675				0.29
Trans-Farnesol	1692	1698				0.99
Benzyl benzoate	1766	1769		8.36		5.52
Guaiazulene	1786	1779		0.83		
Ethyl tetradecanoate	1792	1795		2.42		1.29
2-Ethylhexyl salicylate	1807	1805		1.22		
2-Phenylethyl benzoate	1862	1859		0.56		1.01
Benzyl salicylate	1869	1864		1.77		
Ethyl pentadecanoate	1890	1893		1.07		0.24
9,12,15-Octadecatrienal	2037	2045				0.62
Linalyl anthranilate	2154	2157				0.29
Aliphatic hydrocarbons			36.97	43.17	35.22	19.86

^a^Data for 100% polydimethylsilicone capillary column.

**Table 9:** Results of olfactometric analysis of PE and HPPE powders.

	**Smell descriptors**			
	**Vanillin (sweet vanilla, creamy, chocolate)**	***p*-Vinyl guaiacol (clove, phenolic, peppery, smoky, woody, powdery)**	**Thymol (herbal, thyme, camphor phenolic)**	**Carvacrol (spicy, woody, camphor thymol)**
Powders				
PE	—	—	—	—
PE upper fraction	—	—	—	—
PE lower fraction	—	—	—	—
HPPE	—	—	1	1
HPPE upper fraction	—	—	—	—
HPPE lower fraction	—	—	—	—

Powder dissolved in 20% NaCl				
PE	4	—	—	—
PE upper fraction	10	—	—	—
PE lower fraction	5	—	—	1
HPPE	2	—	—	4
HPPE upper fraction	10	—	2	10
HPPE lower fraction	2	—	10	10

**Table 10:** Antibacterial effect of spray-dried powders.

**Powder**	**Solvent**	***Escherichia coli*** **ATCC 10536**	***Staphylococcus aureus*** **ATCC 6538**	***Streptococcus agalactiae*** **DSM 20565**
		**MIC (mg mL** ^**−1**^ **)**		
PE powder	Sterile ddH_2_O	NAA	NAA	NAA
PE upper fraction powder		NAA	NAA	NAA
PE lower fraction powder		NAA	NAA	25
HPPE powder		NAA	NAA	NAA
HPPE upper fraction powder		NAA	NAA	25
HPPE lower fraction powder		NAA	NAA	25

PE powder	100% DMSO	NAA	NAA	NAA
PE upper fraction powder		NAA	NAA	NAA
PE lower fraction powder		NAA	NAA	NAA
HPPE powder		NAA	NAA	NAA
HPPE upper fraction powder		NAA	NAA	NAA
HPPE lower fraction powder		NAA	NAA	NAA

PE powder	95% ethanol	NAA	NAA	NAA
PE upper fraction powder		NAA	NAA	NAA
PE lower fraction powder		NAA	NAA	NAA
HPPE powder		NAA	NAA	NAA
HPPE upper fraction powder		NAA	NAA	NAA
HPPE lower fraction powder		NAA	NAA	NAA

Abbreviation: NAA, no antibacterial activity in the tested dilutions from 0.39 to 50 mg mL^−1^.

## Data Availability

The data that support the findings of this study are available from the corresponding author upon reasonable request.

## References

[1] Bankova V. (2005). Chemical Diversity of Propolis and the Problem of Standardization. *Journal of Ethnopharmacology*.

[2] Simone-Finstrom M., Spivak M. (2010). Propolis and Bee Health: The Natural History and Significance of Resin Use by Honey Bees. *Apidologie*.

[3] Rojczyk E., Klama-Baryła A., Łabuś W., Wilemska-Kucharzewska K., Kucharzewski M. (2020). Historical and Modern Research on Propolis and Its Application in Wound Healing and Other Fields of Medicine and Contributions by Polish Studies. *Journal of Ethnopharmacology*.

[4] Hossain R., Quispe C., Khan R. A. (2022). Propolis: An Update on Its Chemistry and Pharmacological Applications. *Chinese Medicine*.

[5] Basista K., Filipek B. (2013). Allergic Potential of Propolis - A Literature Review. *Alergia Astma Immunologia*.

[6] Burdock G. A. (1998). Review of the Biological Properties and Toxicity of Bee Propolis (Propolis). *Food and Chemical Toxicology*.

[7] Miłek M., Ciszkowicz E., Tomczyk M. (2022). The Study of Chemical Profile and Antioxidant Properties of Poplar-Type Polish Propolis Considering Local Flora Diversity in Relation to Antibacterial and Anticancer Activities in Human Breast Cancer Cells. *Molecules*.

[8] Przybyłek I., Karpiński T. M. (2019). Antibacterial Properties of Propolis. *Molecules*.

[9] Kubiliene L., Laugaliene V., Pavilonis A. (2015). Alternative Preparation of Propolis Extracts: Comparison of Their Composition and Biological Activities. *BMC Complementary and Alternative Medicine*.

[10] Pobiega K., Kraśniewska K., Derewiaka D., Gniewosz M. (2019). Comparison of the Antimicrobial Activity of Propolis Extracts Obtained by Means of Various Extraction Methods. *Journal of Food Science and Technology*.

[11] Kurek-Górecka A., Rzepecka-Stojko A., Górecki M., Stojko J., Sosada M., Swierczek-Zieba G. (2013). Structure and Antioxidant Activity of Polyphenols Derived From Propolis. *Molecules*.

[12] Ristivojević P., Trifković J., Andrić F., Milojković-Opsenica D. (2015). Poplar-Type Propolis: Chemical Composition, Botanical Origin and Biological Activity. *Natural Product Communications*.

[13] Miłek M., Franke G., Tomczyk M. (2024). The Influence of Geographical Origin on Poplar Propolis Composition and the Impact of Human Microbiota. *Pharmaceuticals*.

[14] Pobiega K., Kraśniewska K., Gniewosz M. (2019). Application of Propolis in Antimicrobial and Antioxidative Protection of Food Quality – A Review. *Trends in Food Science and Technology*.

[15] Mafra J. F., de Santana T. S., Cruz A. I. C. (2022). Influence of Red Propolis on the Physicochemical, Microbiological and Sensory Characteristics of Tilapia (*Oreochromis niloticus*) Salami. *Food Chemistry*.

[16] El-Sakhawy M., Salama A., Mohamed S. A. A. (2024). Propolis Applications in Food Industries and Packaging. *Biomass Conversion and Biorefinery*.

[17] Thamnopoulos I. A. I., Michailidis G. F., Fletouris D. J., Badeka A., Kontominas M. G., Angelidis A. S. (2018). Inhibitory Activity of Propolis Against Listeria Monocytogenes in Milk Stored Under Refrigeration. *Food Microbiology*.

[18] Segueni N., Boutaghane N., Asma S. T. (2023). Review on Propolis Applications in Food Preservation and Active Packaging. *Plants*.

[19] Cortés-Higareda M., de Lorena Ramos-García M., Correa-Pacheco Z. N., Del Río-García J. C., Bautista-Baños S. (2019). Nanostructured Chitosan/Propolis Formulations: Characterization and Effect on the Growth of Aspergillus flavus and Production of Aflatoxins. *Heliyon*.

[20] Bodini R. B., Sobral P. J. A., Favaro-Trindade C. S., Carvalho R. A. (2013). Properties of Gelatin-Based Films With Added Ethanol-Propolis Extract. *LWT-Food Science and Technology*.

[21] Tavares L., Smaoui S., Lima P. S., de Oliveira M. M., Santos L. (2022). Propolis: Encapsulation and Application in the Food and Pharmaceutical Industries. *Trends in Food Science and Technology*.

[22] Marangoni Júnior L., de Avila Gonçalves S., Garcia da Silva R. (2022). Effect of Green Propolis Extract on Functional Properties of Active Pectin-Based Films. *Food Hydrocolloids*.

[23] da Silva F. C., Favaro-Trindade C. S., de Alencar S. M., Thomazini M., Balieiro J. C. C. (2011). Physicochemical Properties, Antioxidant Activity and Stability of Spray-Dried Propolis. *Journal of ApiProduct and ApiMedical Science*.

[24] Busch V. M., Pereyra-Gonzalez A., Šegatin N., Santagapita P. R., Poklar Ulrih N., Buera M. P. (2017). Propolis Encapsulation by Spray Drying: Characterization and Stability. *LWT-Food Science and Technology*.

[25] Baysan U., Elmas F., Koç M. (2019). The Effect of Spray Drying Conditions on Physicochemical Properties of Encapsulated Propolis Powder. *Journal of Food Process Engineering*.

[26] Baysan U., Zungur Bastıoğlu A., Coşkun N. Ö. (2021). The Effect of Coating Material Combination and Encapsulation Method on Propolis Powder Properties. *Powder Technology*.

[27] Dżugan M., Miłek M., Sidor E. (2025). Method of Obtaining a Propolis-Based Food Biopreservative, Polish Patent no. P. 441843.

[28] Kowalska G., Rosicka-Kaczmarek J., Miśkiewicz K. (2021). Influence of Rye Bran Heteropolysaccharides on the Physicochemical and Antioxidant Properties of Honeydew Honey Microcapsules. *Food and Bioproducts Processing*.

[29] Singleton V. L., Rossi J. A. (1965). Colorimetry of Total Phenolics With Phosphomolybdic-Phosphotungstic Acid Reagents. *American Journal of Enology and Viticulture*.

[30] Biju J., Reddy V. R. K., Sulaiman C. T. (2013). Total Phenolics and Flavonoids in Selected Justicia Species. *Journal of Pharmacognosy and Phytochemistry*.

[31] Benzie I. F. F., Strain J. J. (1996). The Ferric Reducing Ability of Plasma (FRAP) as a Measure of “Antioxidant Power”: The FRAP Assay. *Analytical Biochemistry*.

[32] Blois M. S. (1958). Antioxidant Determinations by the Use of a Stable Free Radical. *Nature*.

[33] Re R., Pellegrini N., Proteggente A., Pannala A., Yang M., Rice-Evans C. (1999). Antioxidant Activity Applying an Improved ABTS Radical Cation Decolorization Assay. *Free Radical Biology & Medicine*.

[34] Miłek M., Bonikowski R., Dżugan M. (2024). The Effect of Extraction Conditions on the Chemical Profile of Obtained Raw Poplar Propolis Extract. *Chemical Papers*.

[35] Zaguła G., Fabisiak A., Bajcar M., Czernicka M., Saletnik B., Puchalski C. (2016). Mineral Components Analysis of Selected Dried Herbs. *Econtechmod. An International Quarterly Journal*.

[36] Fratianni F., De Martino L., Melone A., De Feo V., Coppola R., Nazzaro F. (2010). Preservation of Chicken Breast Meat Treated With Thyme and Balm Essential Oils. *Journal of Food Science*.

[37] Boskovic M., Glisic M., Djordjevic J., Vranesevic J., Djordjevic V., Baltic M. Z. (2019). Preservation of Meat and Meat Products Using Nanoencapsulated Thyme and Oregano Essential Oils. *IOP Conference Series: Earth and Environmental Science*.

[38] Asif A., Ibrahim F., Ansari A. (2024). A Systematic Review: Effectiveness of Herbs and Spices as Natural Preservatives to Enhance Meat Shelf-Life. *Journal of Health and Rehabilitation Research*.

[39] Bansal R. A. (2018). Efficacy of Basil Oil in Preservation of Orange Juice. *International Research Journal of Engineering and Technology*.

[40] El-Saadony M. T., Saad A. M., Elakkad H. A. (2022). Flavoring and Extending the Shelf Life of Cucumber Juice With Aroma Compounds-Rich Herbal Extracts at 4 °C Through Controlling Chemical and Microbial Fluctuations. *Saudi Journal of Biological Sciences*.

[41] Lotmani Z., Nadjib Boukhatem M., Boudjema K., Rezzoug H., Benelmouffok A. B., Tomi P. (2024). Commercial Thyme Essential Oil as Natural Beverage Preservative and Molecular Docking Study on its Mode of Action Against Saccharomyces cerevisiae. *Czech Journal of Food Sciences*.

[42] Duque-Soto C., Ruiz-Vargas A., Rueda-Robles A., Quirantes-Piné R., Borrás-Linares I., Lozano-Sánchez J. (2023). Bioactive Potential of Aqueous Phenolic Extracts of Spices for Their Use in the Food Industry—A Systematic Review. *Foods*.

[43] Marcinčáková D., Hudáková N., Miłek M. (2024). Evaluation of the Antioxidant Properties and Biological Effects of a Novel Combined Barberry Root-Propolis Extract on HEK293T Cells. *Pharmaceuticals*.

[44] Đurović S., Kojić I., Radić D. (2024). Chemical Constituents of Stinging Nettle (Urtica dioica L.): A Comprehensive Review on Phenolic and Polyphenolic Compounds and Their Bioactivity. *International Journal of Molecular Sciences*.

[45] Moskwa J., Naliwajko S. K., Markiewicz-Żukowska R. (2020). Chemical Composition of Polish Propolis and Its Antiproliferative Effect in Combination With Bacopa monnieri on Glioblastoma Cell Lines. *Scientific Reports*.

[46] Bankova V., Popova M., Trusheva B. (2014). Propolis Volatile Compounds: Chemical Diversity and Biological Activity: A Review. *Chemistry Central Journal*.

[47] Rathod N. B., Kulawik P., Ozogul F., Regenstein J. M., Ozogul Y. (2021). Biological Activity of Plant-Based Carvacrol and Thymol and Their Impact on Human Health and Food Quality. *Trends in Food Science and Technology*.

[48] Gao T., Zhang Y., Shi J., Mohamed S. R., Xu J., Liu X. (2021). The Antioxidant Guaiacol Exerts Fungicidal Activity Against Fungal Growth and Deoxynivalenol Production in Fusarium graminearum. *Frontiers in Microbiology*.

[49] Guan H., Luo W., Bao B. (2022). A Comprehensive Review of Rosmarinic Acid: From Phytochemistry to Pharmacology and Its New Insight. *Molecules*.

[50] Ivanov M., Kostić M., Stojković D., Soković M. (2022). Rosmarinic Acid–Modes of Antimicrobial and Antibiofilm Activities of a Common Plant Polyphenol. *South African Journal of Botany*.

[51] Kernou O. N., Azzouz Z., Madani K., Rijo P. (2023). Application of Rosmarinic Acid With Its Derivatives in the Treatment of Microbial Pathogens. *Molecules*.

[52] Calatrava E., Rezaei N. (2002). Other Streptococcus Species and Enterococcus. *Encyclopedia of Infection and Immunity*.

[53] Bosio K., Avanzini C., D’Avolio A., Ozino O., Savoia D. (2000). In Vitro Activity of Propolis Against Streptococcus pyogenes. *Letters in Applied Microbiology*.

[54] Zumla A. (2010). Mandell, Douglas, and Bennett's Principles and Practice of Infectious Diseases. *Lancet Infectious Diseases*.

[55] Brouwer S., Rivera-Hernandez T., Curren B. F. (2023). Pathogenesis, Epidemiology and Control of Group a Streptococcus Infection. *Nature Reviews Microbiology*.

[56] Bouzahouane H., Ayari A., Guehria I., Riah O. (2021). Propolis: Antimicrobial Activity and Chemical Composition Analysis. *Journal of Microbiology, Biotechnology and Food Sciences*.

[57] Karanth S., Feng S., Patra D., Pradhan A. K. (2023). Linking Microbial Contamination to Food Spoilage and Food Waste: The Role of Smart Packaging, Spoilage Risk Assessments, and Date Labeling. *Frontiers in Microbiology*.

[58] Lorenzo J. M., Munekata P. E., Dominguez R., Pateiro M., Saraiva J. A., Franco D. (2018). Main Groups of Microorganisms of Relevance for Food Safety and Stability: General Aspects and Overall Description. *Innovative technologies for food preservation*.

[59] Bosica S., Chiaverini A., De Angelis M. E. (2023). Severe Streptococcus equi Subspecies Zooepidemicus Outbreak From Unpasteurized Dairy Product Consumption, Italy. *Emerging Infectious Diseases*.

[60] Erkmen O., Bozoglu T. F. (2016). *Food Microbiology: Principles Into Practice*.

[61] Jansen-Alves C., Fernandes K. F., Crizel-Cardozo M. M., Krumreich F. D., Borges C. D., Zambiazi R. C. (2018). Microencapsulation of Propolis in Protein Matrix Using Spray Drying for Application in Food Systems. *Food and Bioprocess Technology*.

[62] Zhang Q., Yang A., Tan W., Yang W. (2023). Development, Physicochemical Properties, and Antibacterial Activity of Propolis Microcapsules. *Foods*.

[63] Marquele F. D., Stracieri K. M., Fonseca M. J. V., Freitas L. A. P. (2006). Spray-Dried Propolis Extract. I: Physicochemical and Antioxidant Properties. *Pharmazie*.

[64] Tomczyk M., Zaguła G., Dżugan M. (2020). A Simple Method of Enrichment of Honey Powder With Phytochemicals and Its Potential Application in Isotonic Drink Industry. *LWT-Food Science and Technology*.

[65] Baranauskiene R., Venskutonis P. R., Dewettinck K., Verhé R. (2006). Properties of Oregano (*Origanum vulgare* L.), Citronella (*Cymbopogon nardus* G.) and Marjoram (*Majorana hortensis* L.) Flavors Encapsulated Into Milk Protein-Based Matrices. *Food Research International*.

[66] Arana-Sánchez A., Estarrón-Espinosa M., Obledo-Vázquez E. N., Padilla-Camberos E., Silva-Vázquez R., Lugo-Cervantes E. (2010). Antimicrobial and Antioxidant Activities of Mexican Oregano Essential Oils (*Lippia graveolens* H. B. K.) With Different Composition When Microencapsulated in beta-cyclodextrin. *Letters in Applied Microbiology*.

[67] Tomazelli Júnior O., Kuhn F., Padilha P. J. M. (2018). Microencapsulation of Essential Thyme Oil by Spray Drying and Its Antimicrobial Evaluation Against Vibrio alginolyticus and Vibrio parahaemolyticus. *Brazilian Journal of Biology*.

[68] Plati F., Papi R., Paraskevopoulou A. (2021). Characterization of Oregano Essential Oil (*Origanum vulgare* L. subsp. Hirtum) Particles Produced by the Novel Nano Spray Drying Technique. *Foods*.

[69] Kalogeropoulos N., Konteles S., Mourtzinos I., Troullidou E., Chiou A., Karathanos V. T. (2008). Encapsulation of Complex Extracts in *β*-Cyclodextrin: An Application to Propolis Ethanolic Extract. *Planta Medica*.

[70] Vidović S. S., Vladić J. Z., Vaštag Ž. G., Zeković Z. P., Popović L. M. (2014). Maltodextrin as a Carrier of Health Benefit Compounds in Satureja montana Dry Powder Extract Obtained by Spray Drying Technique. *Powder Technology*.

[71] Vincent C., Boerlin P., Daignault D. (2010). Food Reservoir for Escherichia coli Causing Urinary Tract Infections. *Emerging Infectious Diseases*.

[72] Hirose S., Ohya K., Yoshinari T. (2023). Atypical Diarrhoeagenic Escherichia Coli in Milk Related to a Large Foodborne Outbreak. *Epidemiology and Infection*.

[73] Kadariya J., Smith T. C., Thapaliya D. (2014). Staphylococcus aureus and Staphylococcal Food-Borne Disease: An Ongoing Challenge in Public Health. *BioMed Research International*.

[74] Zwe Y. H., Goh Z. H. E., Chau M. L., Aung K. T., Yuk H. G. (2019). Survival of an Emerging Foodborne Pathogen: Group B Streptococcus (GBS) Serotype III Sequence Type (ST) 283—Under Simulated Partial Cooking and Gastric Fluid Conditions. *Food Science and Biotechnology*.

[75] Casquete R., Castro S. M., Jácome S., Teixeira P. (2016). Antimicrobial Activity of Ethanolic Extract of Propolis in “Alheira”, a Fermented Meat Sausage. *Cogent Food and Agriculture*.

[76] Ali F. H., Kassem G. M., Atta-Alla O. A. (2011). Propolis as a Natural Decontaminant and Antioxidant in Fresh Oriental Sausage. *Veterinaria Italiana*.

[77] Vargas-Sánchez R. D., Torrescano-Urrutia G. R., Acedo-Félix E. (2014). Antioxidant and Antimicrobial Activity of Commercial Propolis Extract in Beef Patties. *Journal of Food Science*.

[78] Sagdic O., Silici S., Yetim H. (2007). Fate of *Escherichia* coli and E. coli O157:H7 in Apple Juice Treated With Propolis Extract. *Annals of Microbiology*.

[79] Koç A. N., Silici S., Mutlu-Sariguzel F., Sagdic O., Koc A. (2007). Antifungal Activity of Propolis in Four Different Fruit Juices. *Food Technology and Biotechnology*.

[80] Kahramanoglu I., Usanmaz S. (2017). Effects of Propolis and Black Seed Oil on the Shelf Life of Freshly Squeezed Pomegranate Juice. *Food Science and Nutrition Studies*.

[81] Yang W., Wu Z., Huang Z. Y., Miao X. (2017). Preservation of Orange Juice Using Propolis. *Journal of Food Science and Technology*.

[82] Luis-Villaroya A., Espina L., García-Gonzalo D., Bayarri S., Pérez C., Pagán R. (2015). Bioactive Properties of a Propolis-Based Dietary Supplement and Its Use in Combination With Mild Heat for Apple Juice Preservation. *International Journal of Food Microbiology*.

[83] Jonaidi Jafari N., Kargozari M., Ranjbar R., Rostami H., Hamedi H. (2018). The Effect of Chitosan Coating Incorporated With Ethanolic Extract of Propolis on the Quality of Refrigerated Chicken Fillet. *Journal of Food Processing and Preservation*.

[84] Mahdavi-Roshan M., Gheibi S., Pourfarzad A. (2022). Effect of Propolis Extract as a Natural Preservative on Quality and Shelf Life of Marinated Chicken Breast (Chicken Kebab). *LWT-Food Science and Technology*.

[85] El-Demery M., Elsebaie E., Zidan N., Essa R. (2016). Efficiency of Propolis and Turmeric Powders as Natural Preservatives in Minced Beef. *Journal of Food and Dairy Sciences*.

[86] Gedikoğlu A. (2022). Antimicrobial and Antioxidant Activities of Commercialized Turkish Propolis Extract, and Application to Beef Meatballs. *Turkish Journal of Agriculture-Food Science and Technology*.

[87] Bernardi S., Favaro-Trindade C. S., Trindade M. A., Balieiro J. C. C., Cavenaghi A. D., Contreras-Castilo C. J. (2013). Italian -Type Salami With Propolis as Antioxidant. *Italian Journal of Food Science*.

[88] Gutiérrez-Cortés C., Suarez Mahecha H. (2014). Antimicrobial Activity of Propolis and Its Effect on the Physicochemical and Sensoral Characteristics in Sausages. *Vitae*.

